# Efficacy and safety of ripretinib in Chinese patients with advanced gastrointestinal stromal tumors: a real-world, multicenter, observational study

**DOI:** 10.3389/fonc.2023.1180795

**Published:** 2023-05-18

**Authors:** Weili Yang, Haoran Qian, Litao Yang, Pengfei Wang, Hailong Qian, Binbin Chu, Zhuo Liu, Jingyu Sun, Dan Wu, Lifeng Sun, Wenqiang Zhou, Jingwei Hu, Xiaolei Chen, Chunhui Shou, Lingxiang Ruan, Yunyun Zhang, Jiren Yu

**Affiliations:** ^1^ Department of Gastrointestinal Surgery, The First Affiliated Hospital, Zhejiang University School of Medicine, Hangzhou, Zhejiang, China; ^2^ Department of Gastrointestinal Surgery, Sir Run Run Shaw Hospital, Zhejiang University School of Medicine, Hangzhou, Zhejiang, China; ^3^ Department of Gastric Surgery, Cancer Hospital of the University of Chinese Academy of Sciences (Zhejiang Cancer Hospital), Institute of Basic Medicine and Cancer (IBMC), Chinese Academy of Sciences, Hangzhou, Zhejiang, China; ^4^ Department of Gastrointestinal Surgery, The First Affiliated Hospital of Wenzhou Medical University, Wenzhou, Zhejiang, China; ^5^ Department of Gastrointestinal Surgery, Ningbo Medical Center Lihuili Hospital, Ningbo, Zhejiang, China; ^6^ Department of Geriatrics, Ningbo Mingzhou Hospital, Ningbo, Zhejiang, China; ^7^ Department of Colorectal Surgery, Cancer Hospital of the University of Chinese Academy of Sciences (Zhejiang Cancer Hospital), Institute of Basic Medicine and Cancer (IBMC), Chinese Academy of Sciences, Hangzhou, Zhejiang, China; ^8^ Department of Medical Oncology, Taizhou Municipal Hospital, Taizhou, Zhejiang, China; ^9^ Department of Gastrointestinal Surgery, The Second Affiliated Hospital, Zhejiang University School of Medicine, Hangzhou, Zhejiang, China; ^10^ Department of Colorectal Surgery, The Second Affiliated Hospital, Zhejiang University School of Medicine, Hangzhou, Zhejiang, China; ^11^ Department of Medical Oncology, Taizhou Cancer Hospital, Taizhou, Zhejiang, China; ^12^ Department of Gastrointestinal Surgery, The Second Affiliated Hospital of Wenzhou Medical College, Wenzhou, Zhejiang, China; ^13^ Department of Radiology, The First Affiliated Hospital, Zhejiang University School of Medicine, Hangzhou, Zhejiang, China; ^14^ Medical Affairs Department, Zai Lab (Shanghai) Co., Ltd, Shanghai, China

**Keywords:** Chinese GIST patients, gastrointestinal stromal tumors, ripretinib, real-world, tyrosine kinase inhibitors (TKI)

## Abstract

**Introduction:**

Mutations in KIT proto-oncogene, receptor tyrosine kinase (KIT) and platelet-derived growth factor receptor-α (PDGFRA) render the available tyrosine kinase inhibitors (TKI) ineffective in treating advanced gastrointestinal stromal tumors (GIST). Ripretinib, a broad-spectrum switch-control kinase inhibitor, has shown increased efficacy and manageable safety, but real-world evidence remains scarce. This study evaluates the efficacy and safety of ripretinib among Chinese patients in a real-world setting.

**Methods:**

Advanced GIST patients (N=23) receiving ripretinib following progression on previous lines of TKI treatment were enrolled to determine the efficacy [progression-free survival (PFS) and overall survival (OS)]. Safety was assessed by the incidence and severity of adverse events (AEs). All statistical analyses were performed using SPSS version 20.0 and a p-value of <0.05 was considered significant.

**Results:**

The median PFS (mPFS) of efficacy analysis set (EAS) (N=21) was 7.1 months. mPFS of patients receiving ripretinib following ≤2 lines of previous TKI treatment and ≥3 prior lines of therapy were 7.1 and 9.2 months, respectively. The median OS (mOS) was 12.0 months and shorter interval between the end of the latest TKI and ripretinib therapy was correlated with longer median PFS and OS (p=0.054 and p=0.046), respectively. Alopecia and asthenia were the most common AEs observed.

**Conclusion:**

Compared to previous lines of TKI in advanced GIST patients, ripretinib showed superior efficacy with clinically manageable AEs. Real-world results are comparable to that of phase III INVICTUS study and its Chinese bridging study. Hence, ripretinib can be used for the clinical management of advanced GIST patients.

## 1 Introduction

Gastrointestinal stromal tumors (GISTs) are highly common mesenchymal neoplasms of the gastrointestinal tract responsible for 1% to 2% of malignant gastrointestinal tumors globally (1). Incidence of GIST is estimated to be 1-2 per 100,000 per year worldwide (2), while in China, the crude incidence rate 0.40 per 100,000 person per year has been reported (3). Surgical removal is the standard practice for localized and resectable GIST. However, 40% of the resected patients may relapse or metastasize after surgery (4). KIT proto-oncogene, receptor tyrosine kinase (KIT, ~69%-83%) or platelet-derived growth factor receptor A (PDGFRA, ~5%-10%) oncogene mutations are the primary drivers in GIST pathogenesis. Exon 11 (juxtamembrane domain inhibitory switch) or exon 9 (extracellular domain) are the common primary KIT gene mutations (5), whereas exons 13/14 (cytoplasmic ATP-binding domain) or exons 17/18 (activation loop) are the common secondary KIT mutations (6). Targeting the common driver mutations in these genes using tyrosine kinase inhibitors (TKIs) has become the cornerstone for the treatment of GISTs.

TKIs such as imatinib, sunitinib and regorafenib are currently being used as first, second and third-line treatments, respectively for non-resectable and/or metastatic GIST (7). Imatinib significantly improves the prognosis of GIST patients in the first-line, however ~50% patients with advanced GIST develop into progressive disease by 24 months (8, 9). Sunitinib and regorafenib have shown to improve the outcomes as second- and third-line therapy, respectively, but resistance resulting from secondary mutations in KIT/PDGFRA genes render them ineffective, leading to disease progression after a median progression free survival (mPFS) of 5.6 months and 4.8 months, respectively.

Secondary mutations in the ATP binding domain or activation loop of KIT/PDGFRA hamper the TKI binding sterically, resulting in incomplete inhibition. Both sunitinib and regorafenib are effective against certain secondary mutations but not all (10, 11). Hence, a pressing unmet clinical need exists for the management of advanced GIST to overcome the resistance conferred by secondary mutations. Ripretinib is a novel switch-control kinase inhibitor that broadly inhibits KIT and PDGFRA kinase signaling through a dual mechanism of action. It locks the kinase in an inactive state, prevents downstream signaling and cell proliferation by blocking both switch pocket and activation loop. Dual mechanism of action provides broad inhibition of KIT/PDGFRA wild-type as well as primary and secondary mutations (12, 13). A phase I study of ripretinib showed good tolerability in advanced GIST patients with an objective response rate (ORR) of 11.3% (ranging from 7.2% in ≥fourth-line to 19.4% in second-line) and mPFS ranging between 5.5 months (≥fourth-line therapy) and 10.7 months (second-line therapy) (10). Further, the promising outcomes from global phase III INVICTUS (mPFS: 6.3 months and overall survival (OS): 18.2 months vs placebo) led to its approval by FDA in 2020 as fourth-line treatment option in patients with advanced GIST. A significant improvement in mPFS of patients with various KIT primary and secondary mutations was also noted (7, 12). Further, a phase II Chinese bridging study of INVICTUS evaluating ripretinib as fourth or later-line of treatment in advanced GIST patients showed outcomes comparable to that of INVICTUS study (mPFS-7.2 months; ORR-18.4%) supporting the use of ripretinib in China and is now approved by National Medical Products Administration (NMPA) in treatment of such patients (11). Similarly, another multicenter study conducted in Chinese, Hong Kong and Taiwanese patients with pretreated metastatic GIST showed a mPFS of 6.1 months and response rate of 25% with ripretinib (14).

Though, the structured randomized clinical trials (RCTs) have well established the clinical potential of ripretinib, real-world data on efficacy and safety of ripretinib in China is lacking. Hence, to understand how ripretinib performs in real world scenario, we conducted this real-world, observational study to evaluate the efficacy and safety of ripretinib in Chinese patients with advanced GIST.

## 2 Methods

### 2.1 Study design and patients

This is a real-world, multicenter, observational study aimed to evaluate the efficacy and safety of ripretinib in Chinese patients with advanced GIST who have progressed on previous lines of TKI treatment with imatinib, sunitinib, regorafenib or other TKIs or had documented intolerance to any of TKI treatment despite dose modification. Histologically confirmed advanced GIST patients with Eastern Cooperative Oncology Group (ECOG) performance status of 0–4 who received ripretinib between January 1, 2021 and December 31, 2021 in Zhejiang province of China, were enrolled. All the patients who had at least one imaging assessment for efficacy analysis were included.

### 2.2 Study procedures

Patients were differentiated into full analysis set (FAS) defined as patients treated with ripretinib and efficacy analysis set (EAS) defined as patients with at least 1 efficacy evaluation data. Patients enrolled in EAS were analyzed for efficacy and safety. Safety was analyzed in all patients who have received at least 1 dose of ripretinib. Patients received ripretinib 150 mg once daily continuously in 28-day cycles until progressive disease (PD), intolerable toxicity or economic or other reasons in real-world settings. Relevant data on demographics including physical, clinical and laboratory examination, documented mutation analysis, AEs and dose interruption or reduction were collected.

### 2.3 Outcomes

PFS was the primary efficacy outcome, defined as the time interval between the first dose of ripretinib to PD or death (whichever occurs first). Secondary outcomes included OS (defined as the interval between treatment initiation to death of any cause), ORR (defined as the proportion of patients with a complete response [CR] or partial response [PR] to treatment) and disease control rate (DCR- defined as the percentage of cases with CR, PR and stable disease [SD] ≥4 weeks in patients with evaluable efficacy). Efficacy was evaluated as per the Response Evaluation Criteria in Solid Tumors (RECIST) v1.1 GIST-Specific Standard (15). Safety outcomes included incidence of AEs of any grade, and AEs leading to dose reduction, interruption or discontinuation of ripretinib and death.

PD is defined as at least a 20% increase in the sum of the longest diameter (LD) of target lesions, taking as reference the smallest sum LD recorded since the treatment started or the appearance of one or more new lesions. PR is defined as at least a 30% decrease in the sum of the LD of target lesions, taking as reference the baseline sum LD. SD is defined as neither sufficient shrinkage to qualify for PR nor sufficient increase to qualify for PD, while CR is defined as the disappearance of all target lesions. The definitions of treatment responses were defined as per RECIST v1.1 GIST-Specific Standard (15).

### 2.4 Statistical analyses

Descriptive statistics were used to describe the continuous variables presented as median and range. Percentages and frequencies were used to describe the categorical variables. EAS patients were further grouped based on the number of previous lines of TKI treatment, time interval between the end of the latest TKI and ripretinib therapy and primary KIT/PDGFRA mutations. Comparison between the groups was performed using Cochran-Mantel-Haenszel-χ2 (CMH-χ2) test, Fisher’s exact test or Wilcoxon rank sum test for rank data. Kaplan–Meier method with log-rank test was used to perform survival analysis with 95% confidence interval (CI). A p-value of <0.05 was considered statistically significant. All the statistical analyses were performed using SPSS version 20.0 (IBM Corp., NY, USA).

## 3 Results

### 3.1 Baseline characteristics

**Figure 1:**
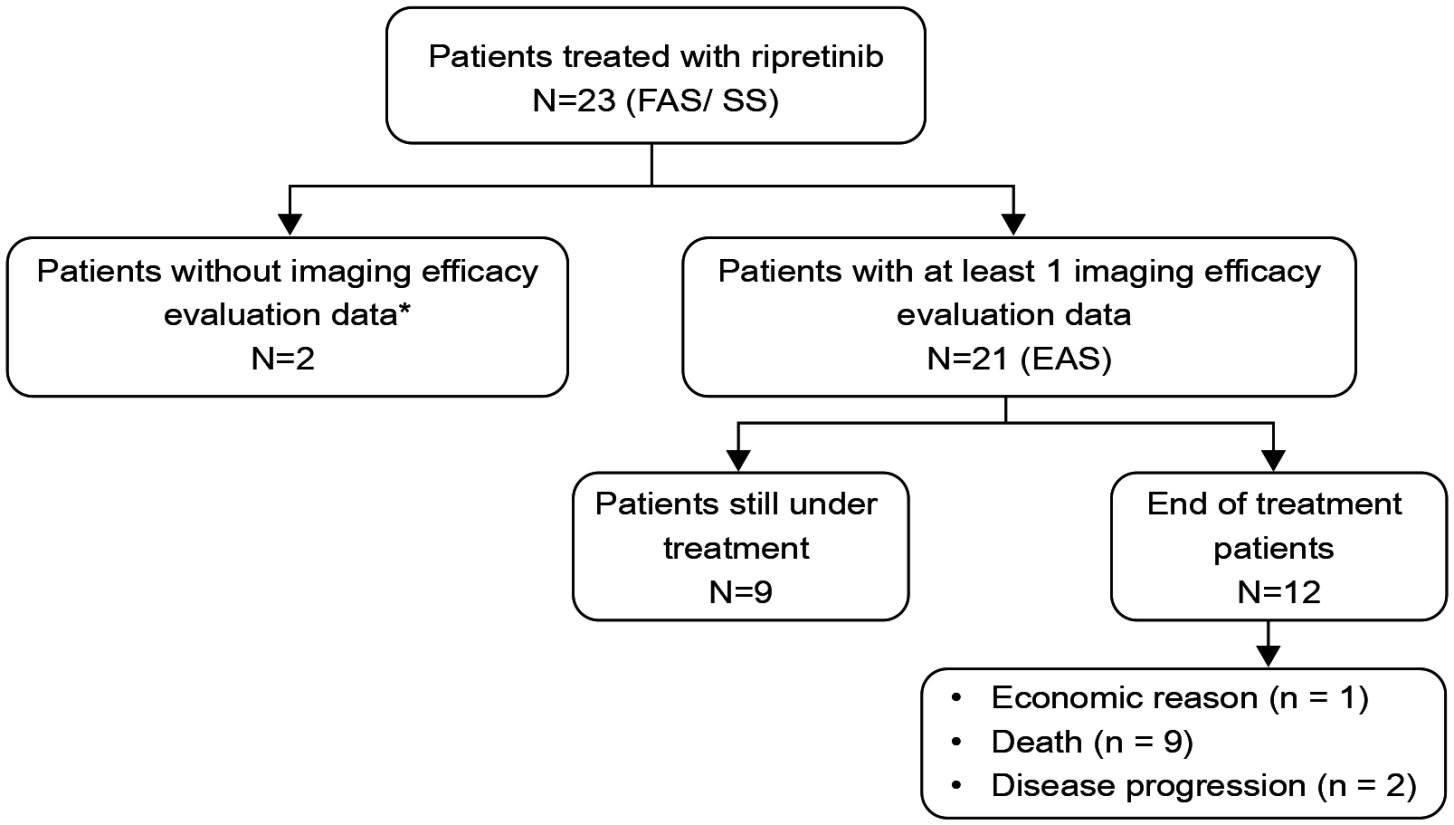
Distribution of study patients who had received ripretinib. FAS, Full analysis set; EAS, efficacy analysis set; SS, safety set. One patient fell off after one month of medication because of long-term bed rest, prominent basic disease and inability to eat; another patient took ripretinib for one and half months and stopped taking it. There were no imaging evaluation materials available for this two patients and were excluded from the efficacy analyses.

A total of 23 patients with advanced GIST were enrolled in the study (FAS), while 21 patients with available imaging results were included in EAS for efficacy analyses. Survival outcomes such as PFS and OS was only calculated for EAS patients for whom efficacy analyses were available (**Figure 1**). The median age of patients receiving ripretinib was 64 years (range, 45–90) and almost half of the patients were aged ≥65 years (n=10; 47.62%). The majority of patients were males (n=13; 61.9%). The site of primary tumor was predominantly the small intestine (n=13; 61.9%) and 47.62% (n=10) of patients had ECOG performance status ≥2. Patients were followed up for 12 months and the median duration of ripretinib treatment was 7.3 months. Most of the patients had received ≥3 previous lines of TKI treatment (n=14; 66.67%) and around 62% of patients (n=13) also presented with secondary mutations in KIT exon 13/14/17/18. Mutation status is a composite of initial diagnosis or after progression on 1^st^ line therapy. Metastases was diagnosed in all the patients at the time of study enrollment with liver being the most common site of metastases (n=16; 76.19%). All the baseline demographic and clinical characteristics are presented in **Table 1**.

**Table 1:** Baseline demographic and clinical characteristics.

Parameter	Efficacy analysis set (EAS) N=21
Age (years)	
Median age at first visit (min, max)	57 (37, 88)
Median age of patients receiving ripretinib (min, max)	64 (45, 90)
Age distribution, n (%)	
<65 years old	11 (52.38)
≥65 years old	10 (47.62)
Sex, n (%)	
Male	13 (61.90)
Female	8 (38.10)
Primary tumor site at first diagnosis, n (%)	
Stomach	5 (23.81)
Small intestine	13 (61.9)
Rectum	2 (9.53)
Other	1 (4.76)
Whether there is metastasis at the first diagnosis, n (%)	
Yes	16 (76.19)
No	5 (23.81)
Initial diagnosis Ki67 index, n (%)	
≤5%	6 (28.57)
>5%	9 (42.86)
Unknown	6 (28.57)
Number of previous treatment lines, n (%)	
≤2 lines	7 (33.33)
≥3lines	14 (66.67)
Median (min, max)	3 (0, 4)
Previous treatment drugs, n (%)	
Imatinib	20 (95.24)
Sunitinib	18 (85.71)
Regorafenib	14 (66.67)
Avapritinib	2 (9.52)
Other	1 (4.76)
Previous duration of sunitinib, n (%)	
≤6months	6 (35.29)
>6months	11 (64.71)
Previous duration of regorafenib, n (%)	
≤6 months	9 (69.23)
>6 months	4 (30.77)
ECOG performance score at study enrolment, n (%)	
0	3 (14.28)
1	8 (38.10)
2	8 (38.10)
3	1 (4.76)
4	1 (4.76)
Maximum lesion size at study enrolment, n (%)	
≤10cm	17 (80.95)
>10cm	4 (19.05)
Number of previous operations at the time of study enrolment, n (%)	
0	1 (4.76)
1	13 (61.90)
2	3 (14.29)
>2	4 (19.05)
Presence of metastases at the time of study enrolment, n (%)	
Yes	21 (100)
No	0
Number of metastatic organs at the time of study enrolment, n (%)	
≤2	13 (61.90)
≥3	8 (38.10)
Site of metastasis at study enrolment, n (%)	
Liver	16 (76.19)
Peritoneum	3 (14.29)
Other	16 (76.19)
Number of metastatic foci, n (%)	
1	9 (42.86%)
2	5 (23.81%)
3	5 (23.81%)
4	2 (9.52%)
Tumor mutation*, n (%)	
KIT exon 9	8 (38.10)
KIT exon 11	8 (38.10)
KIT exon 13/14/17/18	13 (61.90)
PDGFRA exon 18	1 (4.76)
Unknown	1 (4.76)

*One patient may have multiple mutations at the same time; As long as a certain mutation is met, it will be included in a mutation classification. Patients may be included in different mutation classifications at the same time. Therefore, the proportion of patients of various types is more than 100%. ECOG, Eastern Cooperative Oncology Group; PDGFRA, Platelet-derived growth factor receptor A; CI, Confidence interval.

### 3.2 Efficacy

#### 3.2.1 Primary endpoint

**Figure 2:**
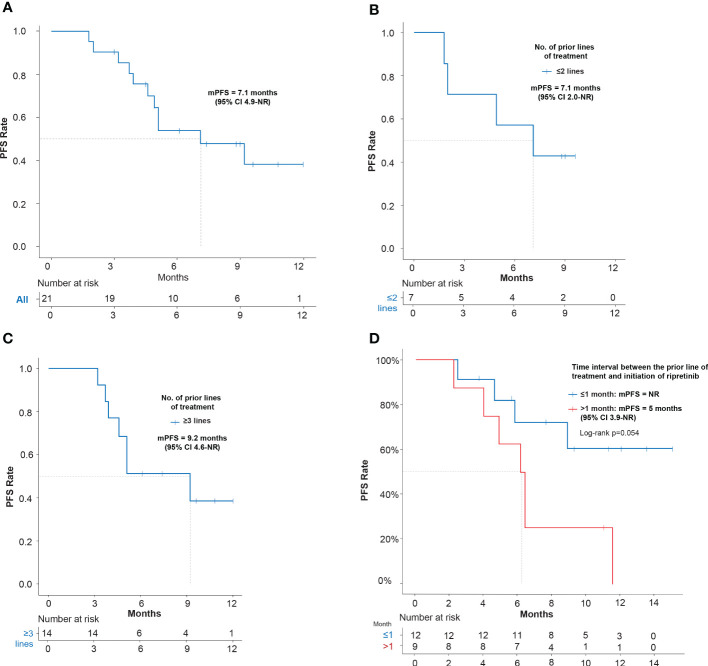
Kaplan–Meier estimates of efficacy in terms of PFS of ripretinib treatment in advanced GIST patients. **(A)** PFS of EAS patients receiving ripretinib; **(B)** PFS of patients receiving ripretinib following ≤2 prior lines of treatment; **(C)** PFS of patients receiving ripretinib following ≥3 prior lines of treatment; **(D)** PFS of EAS patients stratified based on the time interval between the end of the latest TKI and ripretinib therapy. One patient was not included in the time interval stratification analysis, since the patient had received ripretinib regimen as frontline treatment. Censoring events are denoted by crosses. PFS, Progression free survival; PD, Progressive disease; EAS, Efficacy analysis set.

In EAS (N=21), mPFS of 7.1 months (95% CI: 4.9–NR) was observed (**Figure 2A**). The mPFS was 7.1 months (95% CI: 2.0–NR) in patients who had ≤2 prior lines (n=7; 33.3%) and 9.2 months (95% CI, 4.6–NR) in patients with ≥3 prior lines (n=14; 66.7%) (**Figures 2B, C**). Patients were also stratified and analyzed based on the time interval between the end of the latest TKI and ripretinib therapy. A better trend of improvement (p=0.054) in mPFS was observed in patients switching to ripretinib within the time interval of ≤1 month who did not reach the mPFS, while the patients switching to ripretinib at a time interval of >1 month who had a mPFS of 5.0 months (95% CI: 3.9–NR) (**Figure 2D**). When mutations were taken into account, patients with KIT exon 11 mutation showed a mPFS of 7.1 months (95% CI: 5.1-NR), while it was 3.9 months (95% CI: 3.7-NR) in patients with KIT exon 9 mutation (**Table 2**).

**Table 2:** Stratified analysis on the efficacy of ripretinib based on number of treatment lines and different gene mutations.

Subgroup	No. of cases	PR, n (%)	SD, n (%)	PD, n (%)	ORR, n (%)	DCR, n (%)	mPFS, month (95% CI)
≤2 previous lines of treatment	7	1 (14.29)	3 (42.85)	3 (42.85)	1 (14.29)	4 (57.14)	7.1 (2.0, NR)
≥3 previous lines of treatment	14	1 (7.14)	13 (92.86)	0	1 (7.14)	14 (100.00)	9.2 (4.6, NR)
Ripretinib switch in a time interval of ≤1 month	12	2 (16.67)	9 (75.00)	1 (8.33)	2 (16.67)	11 (91.67)	NR
Ripretinib switch in a time interval of >1 month	8	0	6 (75.00)	2 (25.00)	0	6 (75.00)	5.0 (3.9, NR)
KIT exon 11	8	2 (25.00)	6 (75.00)	0	2 (25.00)	8 (100.00)	7.1 (5.1, NR)
KIT exon 9	8	0	5 (62.50)	3 (37.5)	0	5 (62.50)	3.9 (3.7, NR)
PDGFRA mutation	1	0	1 (100.00)	0	0	1 (100.00)	NR
Genotype unknown	1	0	1 (100.00)	0	0	1 (100.00)	NR

PDGFRA, Platelet-derived growth factor receptor A; CI, Confidence interval; NR, Not reached; PR, Partial response; SD, Stable disease; PD, Progressive disease; ORR, Objective response rate; DCR, Disease control rate; mPFS, Median progression free survival.

#### 3.2.2 Secondary endpoints

##### 3.2.2.1 Overall survival

**Figure 3:**
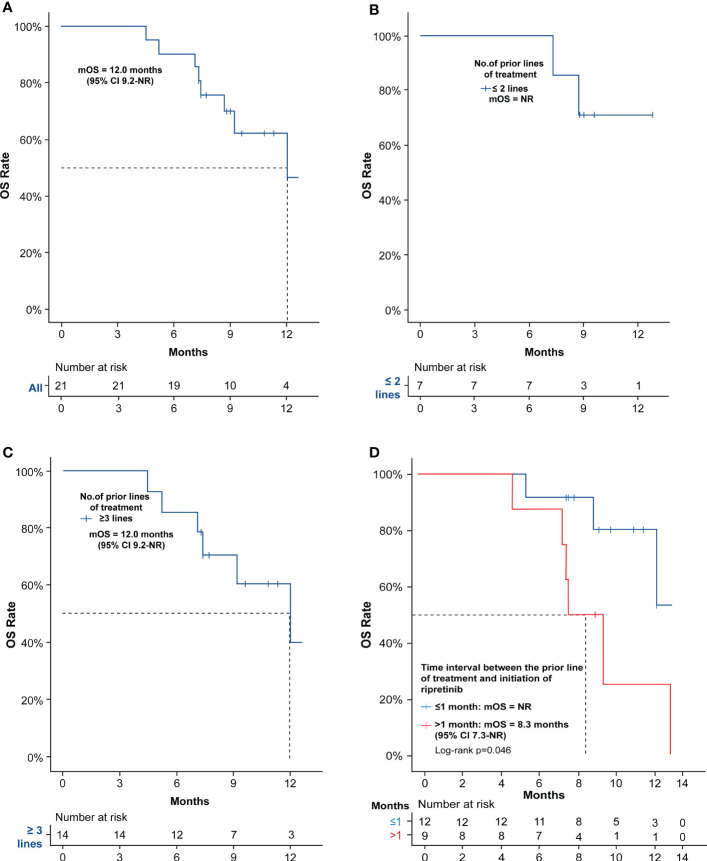
Kaplan–Meier estimates of efficacy in terms of OS of ripretinib treatment in advanced GIST patients. **(A)** OS of EAS patients receiving ripretinib; **(B)** OS of patients receiving ripretinib following ≤2 prior lines of treatment; **(C)** OS of patients receiving ripretinib following ≥3 prior lines of treatment; **(D)** OS of EAS patients stratified based on the time interval between the end of the latest TKI and ripretinib therapy. One patient was not included in the time interval stratification analysis, since the patient had received ripretinib regimen as frontline treatment. Censoring events are denoted by crosses. OS, Overall survival; PD, Progressive disease; EAS, efficacy analysis set.

All the patients who progressed on ripretinib 150 mg QD did not receive dose escalation to ripretinib 150 mg b.i.d., and the mOS was 12 months (95% CI: 9.2–NR) (**Figure 3A**). Patients who underwent ≤2 prior lines of treatment did not reach mOS while patients with ≥3 prior lines of treatment had a mOS of 12 months (95% CI: 9.2–NR) (**Figures 3B, C**). When the OS was assessed according to the time interval between the end of the latest TKI and ripretinib therapy, patients who switched to ripretinib after a gap of >1 month had a mOS of 8.3 months (95% CI: 7.3–NR), while mOS was not reached for patients switching within ≤1 month and the differences in mOS was statistically significant (p <0.05) (**Figure 3D**).

##### 3.2.2.2 ORR and DCR

An ORR of 9.52% and DCR of 85.71% were achieved with treatment on ripretinib in EAS set. While none of the patients treated with ripretinib achieved CR, 2 (9.52%), 16 (76.19%) and 3 (14.28%) patients had PR, SD and PD, respectively. Treatment response was also analyzed in patients stratified based on the number of prior lines of treatment. Patients who had received ≤2 prior lines of treatment showed an ORR of 14.29% and DCR of 57.14% while the patients who received ≥3 prior lines of treatment had an ORR of 7.14% and DCR of 100% with ripretinib treatment. Patients with KIT exon 11 mutations (n=8) achieved an ORR of 25% and a DCR of 100%. ORR was not observed in patients with KIT exon 9 mutations (n=8) but DCR was 62.5% (**Table 2**).

### 3.3 Adverse events

All the patients enrolled (safety set, N=23) were analyzed for AEs and the incidence is summarized in **Table 3**. Alopecia and asthenia were the most common AEs of any grade (n=7 each; 30.43%) and almost were grade 1-2 (n=7; 30.43% and n=6; 26.09%, respectively). Abdominal pain and decreased lymphocyte count (n=2 each; 8.70%) were the most common grade 3 AEs observed. AEs leading to ripretinib dose reduction were observed in 2 patients (8.70%). No deaths due to AEs or new safety signals were reported (**Table 3**).

**Table 3:** Adverse events (safety set).

	N=23		
Adverse events, n (%)	Any grade	Grade 1-2	Grade 3
Alopecia	7 (30.43)	7 (30.43)	0
Asthenia	7 (30.43)	6 (26.09)	1 (4.35)
Palmar–plantar erythrodysesthesia syndrome	3 (13.04)	3 (13.04)	0
Hypertension	3 (13.04)	3 (13.04)	0
Diarrhoea	3 (13.04)	2 (8.70)	1 (4.35)
Abdominal pain	3 (13.04)	1 (4.35)	2 (8.70)
Decreased lymphocyte count	3 (13.04)	1 (4.35)	2 (8.70)
Decreased appetite	2 (8.70)	2 (8.70)	0
Hyperpigmentation of skin	2 (8.70)	2 (8.70)	0
Myalgia	2 (8.70)	1 (4.35)	1 (4.35)
Vomiting	1 (4.35)	1 (4.35)	0
Rash	1 (4.35)	1 (4.35)	0
Palpitation	1 (4.35)	1 (4.35)	0
Nausea	1 (4.35)	1 (4.35)	0
Perianal pain	1 (4.35)	1 (4.35)	0
Limb pain	1 (4.35)	1 (4.35)	0
Skin Itch	1 (4.35)	1 (4.35)	0
Skin induration	1 (4.35)	1 (4.35)	0
Intestinal obstruction	1 (4.35)	0	1 (4.35)
AEs leading to dose adjustment			
Type of AE, n (%)	**Total no. of cases**		
Dose reduction due to any AE	2 (8.70)		
Grade 3 myalgia	1 (4.35)		
Grade 3 perianal pain	1 (4.35)		

The number of cases is the number of patients. AE, Adverse event.

## 4 Discussion

Real-world studies play a crucial role in generating data that are important in determining the efficacy of drugs outside of the tightly controlled conditions of RCTs. Our multicenter investigation provides one of the preliminary observations on the efficacy of ripretinib in GIST patients in a real-world clinical setting in China. Ripretinib is currently the only drug indicated for the fourth-line treatment of advanced GISTs. Recently concluded global phase III INVICTUS pivotal study showed a mPFS of 6.3 months and ORR of 11.8% with ripretinib (12). A phase II Chinese bridging study of ripretinib GIST patients showed similar mPFS of 7.2 months and ORR of 18.4% (11). Also, mPFS of 6.1 months was reported on ripretinib treatment among Taiwan and Hong Kong patients (14). These results were supplemented by findings (mPFS: 7.1 months; mOS:12 months; ORR: 9.52%) from this real-world study. Further, when stratified based on previous lines of treatment, mPFS of 9.2 months and ORR of 7.14% was observed in patients who had ≥3 lines of prior treatment which were better than those observed with historical 2^nd^ line sunitinib (5.6 months; ORR 6.8%), 3^rd^ line regorafenib (4.8 months; ORR 4.5%), 3^rd^ line imatinib (1.8 months, ORR 0%) and comparable with other studies on ripretinib, showing superior efficacy outcomes with late line treatment of GIST with ripretinib compared to the other TKIs available (16–20). Notably, As in the phase III INVICTUS study, the mPFS was 1 month in the placebo group, an additional 4.6 months of prolonged mPFS was achieved after crossover to ripretinib group (16, 21). Our study also revealed that patients who had switched to ripretinib in ≤1 month from the latest prior treatment, did not reach mPFS (vs 5.0 months in patients switching >1 month) clearly indicating that early initiation of ripretinib after failing on previous therapy was associated with better outcomes.

OS is the gold standard efficacy outcome, which was 14.2 months (95% CI, 7.2–not estimable) in patients randomized to ripretinib 150 mg QD with PD and not receiving intrapatient dose escalation to 150 mg b.i.d. in phase III INVICTUS study (12). Our study reported a similar mOS of 12 months (95% CI: 9.2 – NR) compared to INVICTUS study with patients receiving only ripretinib 150 mg QD. No dose escalation therapy was performed since the patients were constrained by economic factors. However the mOS may be affected by factors such as primary tumor site, mutations and ECOG performance score (22, 23). Non-gastric GISTs are known to be associated with poor outcomes (24). Small intestine (61.9%) was the predominant primary tumor site in this study, followed by gastric (23.81%) tumor which is similar to other ripretinib intervention studies in advanced GIST patients (11, 14). INVICTUS study had higher rate of gastric (47%) tumor patients than in small intestine (26%) (12). Moreover, KIT exon 9 mutation rate was only 17% in INVICTUS study (12) compared to 38.1% in our study. KIT exon 9 mutations are characterized by A502_Y503 codon repetition which are mostly found only in intestinal GIST (25). This often leads to a more aggressive clinical phenotype predominantly occurring in male population. When ECOG score was analyzed, both INVICTUS and the recently reported compassionate-use basis study on ripretinib, included patients with an ECOG score of 0-2 (12, 14). But in our study, 47.62% of patients had an ECOG score of ≥2 indicating a more severe disease. ECOG PS has been shown to have a prognostic value and hence ECOG framework may form the basis of risk stratification of survival in patients with advanced cancer (23). Collectively, small intestine being the predominant primary tumor site, higher KIT exon 9 mutation rate, and a greater percentage of patients with a higher ECOG PS score could have amplified the tumor severity, leading to a shorter mOS in this real-world study. In phase III INVICTUS study, the mOS of patients on placebo without crossover was only 1.8 months (95%CI 0.9-4.9), while in patients with crossover it was 11.6 months (95% CI 6.3-NE).^12^ In our study, we noted that the mOS was not reached in patients switching to ripretinib in shorter interval of ≤1 month. Though this finding cannot be effectively compared with the available data, it shows preliminary evidence on the significant benefit of early ripretinib switching in advanced GIST patients.

The majority of patients in this study achieved SD (76.19%) rather than PR. This observation has drawn parallels with the INVICTUS study where most of the GIST patients achieved SD (66%). Though none of the patients achieved a CR, SD observed in GIST patients is considered as an important marker of therapeutic benefit (26, 27). INVICTUS study set a predefined ORR of 22% but reached only 11.8%, which is similar to the observations of this study (9.52%). As per the RECIST v1.1, tumor control is of paramount importance in advanced GIST rather than a response (15). Hence, the DCR rate of 85.71% observed in our study suggests a high clinical value of ripretinib application in the management of advanced GIST.

In the current study, ripretinib was found be effective in overall GIST patient population (mPFS: 7.1 months; DCR: 85.71%) as well in patients harboring KIT exon 11 mutations (mPFS: 7.1 months; DCR: 100%), KIT exon 9 mutations (mPFS: 3.9 months; DCR: 62.5%) and PDGFRA mutations (mPFS: NR; DCR: 100%). Since the reports of ripretinib efficacy based on KIT mutations are scarce, it is difficult to accomplish the clinical benefit of ripretinib only based on the presence of mutations (7). In addition, the small sample size of this study makes it difficult to conclude the ripretinib efficacy based on the type of KIT mutations. Hence, studies with larger real-world samples based on mutation types are needed to further corroborate ripretinib efficacy outcomes in accordance with different mutation patterns.

The safety results from our study were also in line with the published literature. Treatment with ripretinib was well tolerated in our study, which was analogous to INVICTUS and other ripretinib studies. Alopecia was the most common any grade AE reported in our study (30.4%) which was also the case with the global INVICTUS trial (49%) and its Chinese bridging study (43.6%) and another multicenter study in Taiwan and Hong Kong (55%) (11, 12, 14). Ripretinib activity on target kinases and its effects on associated downstream pathways might play a role in hair fall, but a clear understanding of its association is not yet identified (28, 29). General asthenia (30.43%) was the second leading AE reported in this study while it was one of the most common treatment-related adverse events (TRAE) (23.1%) in the Chinese bridging study but not in other ripretinib studies (11). While the incidence of Palmar–plantar erythrodysesthesia syndrome has been reported in the range of 20-25% in previous ripretinib studies, an incidence of 13% was reported in our study (12). Abdominal pain and decreased lymphocyte counts were the most frequent grade 3 AEs (8.7% each) while it was increased lipase (5%) in INVICTUS study (12). Anaemia and diarrhea (5% each) were the frequent grade 3 AE observed in another multicentre study (14). Overall, the AEs were clinically manageable and there were no grade 4 or 5 treatment emergent adverse events (TEAEs) reported. No new safety signals were recorded in this real-world study.

Besides the general limitation associated with real world studies like the potential for any bias and confounding factors that are generally controlled in RCTs. As this study is a real-world, observational study and not a randomized nature, there may be selection bias in the enrollment of patients. Moreover, demographic, social and economic factors might have acted as barrier of patient participation which might also have influenced the selection bias of the patients enrolled in the study. The main limitation of our study was its modest sample size and relatively short follow-up because of which caution should be exercised while comparing the results from our study with the clinical trials.

## 5 Conclusion

This study evaluated the efficacy and safety of ripretinib in advanced GIST patients following progression on prior TKIs, in a real-world setting in China. The results demonstrated that the efficacy and safety of ripretinib were consistent with those observed in global RCT and Chinese bridging study. This study also showed improved efficacy outcomes in patients for whom ripretinib was initiated early (≤1 month) following progression on previous therapy. Earlier switch to ripretinib appears to benefit the clinical management of patients with advanced refractory GIST progressing on previous TKI, which might improve the survival outcomes in these patients. However, further studies with larger sample sizes are warranted to validate the benefits of early ripretinib switching.

## Data availability statement

The original contributions presented in the study are included in the article/supplementary material. Further inquiries can be directed to the corresponding author.

## Ethics statement

The studies involving human participants were reviewed and approved by institutional review boards (Clinical Research Ethics Committee of the First Affiliated Hospital, College of Medicine, Zhejiang University). The patients/participants provided their written informed consent to participate in this study.

## Author contributions

WY: Conceptualization, Data curation, Analysis, Investigation, Methodology, Writing – original draft. HRQ: Data collection, Methodology, Writing – original draft. LY: Data curation, Visualization, Writing – original draft. PW: Primary data collection, Methodology. HLQ: Methodology, Data review. BC: Writing review and editing, Methodology. ZL: Primary data collection, Writing - original draft. JS: Methodology, Data curation, Data review. DW: Writing - original draft, Writing review and editing. LS: Data curation, Visualization. WZ: Data review, Data validation. JH: Methodology, Writing - review & editing. XC: Methodology, Supervision, Writing - Original draft. CS: Conceptualization, Data analysis, Investigation. LR: Data analysis, Supervision. YZ: Writing - original draft, Writing review and editing. JY: Conceptualization, Data review, Investigation, Methodology, Investigation, Visualization, Supervision, Writing – original draft, Writing – review & editing. The work reported in the paper has been performed by the authors, unless clearly specified in the text. All authors contributed to the article and approved the submitted version.
